# Upstream molecular signaling pathways of p27(Kip1) expression: Effects of 4-hydroxytamoxifen, dexamethasone, and retinoic acids

**DOI:** 10.1186/1475-2867-10-3

**Published:** 2010-02-19

**Authors:** Isao Eto

**Affiliations:** 1 Department of Nutrition Sciences, University of Alabama at Birmingham, Birmingham, Alabama, USA

## Abstract

**Background:**

p27(Kip1) is a cyclin-dependent kinase inhibitor that inhibits G1-to-S phase transition of the cell cycle. It is known that a relatively large number of nutritional and chemopreventive anti-cancer agents specifically up-regulate expression of p27 without directly affecting the expression of other G1-to-S phase cell cycle regulatory proteins including p21(Cip1Waf1). However, the upstream molecular signaling pathways of how these agents up-regulate the expression of p27 have not been well characterized. The objective of this study was to identify such pathways in human breast cancer cells *in vitro *using 4-hydroxytamoxifen, dexamethasone, and various retinoic acids as examples of such anti-cancer agents.

**Results:**

Experimental evidence presented in the first half of this report was obtained by transfecting human breast cancer cells *in vitro *with proximal upstream region of *p27 *gene-luciferase reporter plasmids. 1) The evidence indicated that 4-hydroxytamoxifen, dexamethasone, and various retinoic acids up-regulated expression of p27 in both estrogen receptor-positive and negative human breast cancer cells *in vitro*. 2) The degree of up-regulation of p27 expression by these anti-cancer agents in human breast cancer cells *in vitro *linearly correlated with the degree of inhibition of methylnitrosourea (MNU)-induced rat mammary adenocarcinoma *in vivo*. 3) Lastly, up-regulation of the expression of p27 was likely due to the activation of translation initiation rather than transcription of *p27 *gene. The experimental evidence presented in the second half of this report was obtained by a combination of Western immunoblot analysis and transfection analysis. It indicated that 4-hydroxytamoxifen and dexamethasone up-regulated expression of p27 by down-regulating phosphorylation of eukaryotic translation initiation factor 4E (eIF4E)-binding protein 1 (4E-BP1) at Ser65 and this phosphorylation was likely to be mediated by upstream receptor tyrosine kinases/phosphoinositide-3-kinase/Akt/5'-AMP-activated protein kinase/mammalian target of rapamycin (RTKs/PI3K/Akt/AMPK/mTOR) protein kinase signaling pathways. Retinoic acids up-regulated expression of p27 without using either 4E-BP1 or RTKs/PI3K/Akt/AMPK/mTOR protein kinase signaling pathways.

**Conclusions:**

4-Hydroxytamoxifen and dexamethasone up-regulated translation initiation of p27 by down-regulating 4E-BP1 phosphorylated at Ser65 and this down-regulation seemed to be mediated by upstream RTKs/PI3K/Akt/AMPK/mTOR protein kinase signaling pathways. Retinoic acids also up-regulated translation initiation of p27, but without using any of these pathways.

## Background

Cyclin-dependent kinases (CDKs), together with cyclins, their regulatory subunits, govern cell cycle progression in eukaryotic cells. p27(Kip1) is a member of a family of CDK inhibitors (CDIs) that bind to cyclin/CDK complexes and arrest cell cycle progression from G1 to S phase.

In early G1 phase, mitogens increase D-type cyclins, which bind and activate CDK4 and CDK6 [see reference [1] for an excellent review]. Subsequent activation of cyclin E and cyclin A/CDK2 complexes regulate S phase entry and progression. Two families of CDIs regulate the cyclin/CDK complexes [1-6], namely (a) the inhibitor of CDK4 (INK4) family members and (b) members of kinase inhibitor protein family, p27(Kip1), p57(Kip2) and p21(Cip1Waf1), which bind and inhibit cyclin E and cyclin A-bound CDK2. Although p27 and p21 are major inhibitors of CDK2, they also promote G1 progression by facilitating the assembly of cyclin D/CDK4 and cyclin D/CDK6 complexes [7,8].

It is known that a relatively large number of nutritional and chemopreventive anti-cancer agents specifically up-regulate expression of p27 in eukaryotic cells without directly affecting other G1-to-S phase cell cycle regulatory proteins including INK4s, p57(Kip2), p21(Cip1Waf1), D-type cyclins, cyclin E, cyclin A, CDK2, CDK4 and CDK6 [9,10]. For example, retinoic acids (e.g., all-*trans*, 9-*cis*, and 13-*cis*) and dexamethasone specifically up-regulated expression of p27 in promotion-sensitive (P+) JB6 mouse epidermal cells *in vitro *without affecting cyclin D1, cyclin A and p21 [10]. Also, 4-hydroxytamoxifen (but not tamoxifen), genistein and daidzein (but not genistin), curcumin, taxifolin, retinoic acids (e.g., all-*trans *and 9-*cis*) and dexamethasone up-regulated expression of p27 in estrogen receptor-positive human MCF7 breast cancer cells *in vitro *[10]. Similarly, 4-hydroxytamoxifen (but not tamoxifen), genistein and daidzein (but not genistin), resveratrol, retinoic acids (e.g., all-*trans*, 9-*cis*, and 13-*cis*) and dexamethasone up-regulated expression of p27 in estrogen receptor-negative MDA-MB-231 human breast cancer cells *in vitro *[10]. Additionally, numerous other nutritional and chemopreventive anti-cancer agents up-regulated expression of p27 in MDA-MB-231 cells [10].

Despite all this information, however, very little is known about the upstream molecular signaling pathways of how these anti-cancer agents up-regulate the expression of p27. According to Slingerland, Hengst and other investigators [1,11,12], p27 expression is believed to be regulated at different levels including transcriptional [13-16], translational [11,17-19], and post-translational mechanisms including ubiquitin-proteasome-induced degradation [20-23], complex association [24], subcellular localization [25-30], and protein phosphorylation [12,30,31].

Previously, we identified four different upstream molecular signaling pathways of p27 expression using p27-luciferase reporter plasmids and numerous specific inhibitors and stimulators of p27 expression [10]. (We will call these four pathways as pathway #1, #2, #3 and #4.). This approach was very efficient and sensitive in identifying upstream molecular signaling pathways of p27 expression, but it had a major drawback; namely, it could not tell which specific anti-cancer agent uses which specific pathway to up-regulate p27 expression. To address this question, Western immunoblot analysis, although cumbersome and not as sensitive as p27-luciferase reporter assays, must have been performed. The objective of the present study, therefore, was to perform Western immunoblot analysis using 4-hydroxytamoxifen, dexamethasone, and retinoic acids as examples of anti-cancer agents to identify which specific upstream molecular signaling pathway each one of these anti-cancer agents uses to up-regulate the expression of p27 in human breast cancer cells *in vitro*.

The results indicated that 4-hydroxytamoxifen and dexamethasone up-regulated translation initiation of p27 by down-regulating the phosphorylation of eukaryotic translation initiation factor 4E (eIF4E)-binding protein 1 (4E-BP1). The phosphorylation of 4E-BP1 seemed to be down-regulated by upstream mTOR protein kinase pathways including (a) receptor tyrosine kinases (RTKs)/phosphoinositide-3-kinase (PI3K)/Akt and (b) 5'-AMP-activated protein kinase (AMPK) and then tuberous sclerosis complex (TSC)/mammalian target of rapamycin (mTOR). Retinoic acids also up-regulated translation initiation of p27, but they did so without using any of these pathways including 4E-BP1.

## Results

### 4-Hydroxytamoxifen, dexamethasone, all-trans-retinoic acid and 9-cis-retinoic acid up-regulated expression of p27 in both estrogen receptor-positive and -negative human breast cancer cells in vitro

**Figure 1:**
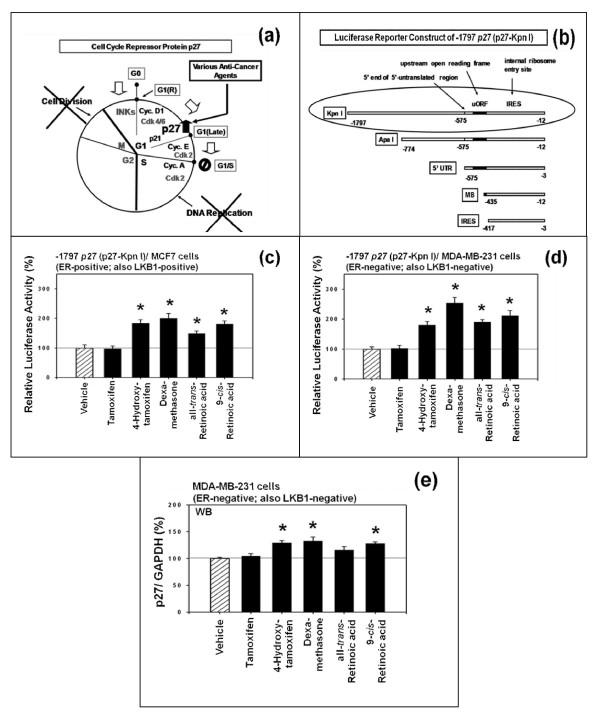
**4-Hydroxytamoxifen, dexamethasone, all-trans-retinoic acid and 9-cis-retinoic acid up-regulate expression of p27 in both estrogen receptor-positive and -negative human breast cancer cells in vitro**. (a) Outline of how various anti-cancer agents specifically up-regulate expression of p27 and arrest the progression of cell cycle from G1 to S phase. (b) Schematic drawing of the luciferase reporter plasmid containing proximal 5'-upstream region (-1797) of the *p27 *gene (-1797 p27 (p27-Kpn I)). 4-Hydroxytamoxifen (but not tamoxifen), dexamethasone, all-*trans*-retinoic acid and 9-*cis*-retinoic acid up-regulated relative luciferase activity of p27 in (c) estrogen receptor (ER)-positive MCF7 and (d) estrogen receptor (ER)-negative MDA-MB-231 human breast cancer cells *in vitro*. (e) Western immunoblot analysis of the expression of p27 protein in estrogen receptor (ER)-negative MDA-MB-231 human breast cancer cells *in vitro*. In all experiments, the cells were exposed to 1 μM each of tamoxifen, 4-hydroxytamoxifen, dexamethasone, all-*trans*-retinoic acid or 9-*cis*-retinoic acid for 24 hours. All assays were performed in triplicates and repeated three times.

The diagram in Figure 1a shows the outline of how various anti-cancer agents specifically up-regulate expression of p27 and arrest cell cycle progression from G1 to S phase. The upstream molecular signaling pathways of how these anti-cancer agents up-regulate the expression of p27 was investigated using a p27-luciferase reporter plasmid containing proximal upstream region (-1797) of *p27 *gene (p27-Kpn I) (Figure 1b) [32]. This plasmid was transfected into the estrogen receptor (ER) - positive as well as negative human breast cancer cells *in vitro *and then the transfected cells were exposed to 1 μM each of the following five different anti-cancer agents, namely tamoxifen, 4-hydroxytamoxifen, dexamethasone, all-*trans *-retinoic acid (atRA), and 9-*cis-*retinoic acid (9cRA) for 24 hours. The results (Figure 1c and 1d) indicated first that tamoxifen did not up-regulate the expression of p27 in both MDA-MB-231 and MCF7 cells, but other four anti-cancer agents up-regulated the expression of p27 in both ER-positive (also LKB1-positive) and ER-negative (also LKB1-negative) human breast cancer cells in *vitro*. Next, expression of p27 protein in ER-negative MDA-MB-231 cells was examined by Western immunoblot analysis. The results (Figure 1e) indicated that tamoxifen and all-*trans*-retinoic acid (atRA) did not up-regulate the expression of p27 protein, but 4-hydroxitamoxifen, dexamethasone and 9-*cis*-retinoic acid (9cRA) did. It should be noted that, although all-*trans*-retinoic acid (atRA) did not up-regulate the expression of p27 protein in a statistically significant manner, average expression of p27 protein tended to be higher in the presence of all-*trans*-retinoic acid (atRA) than in the absence of all-*trans*-retinoic acid (atRA).

In summary, these results suggested that 4-hydroxytamoxifen (but not tamoxifen), dexamethasone, 9-*cis*-retinoic acid (9cRA) and probably all-*trans*-retinoic acid (atRA) up-regulated the expression of p27 in both ER-positive and negative human breast cancer cells *in vitro *(Figures 1c, 1d and 1e).

### The degree of up-regulation of p27 in human breast cancer cells in vitro linearly correlates with the degree of inhibition of methylnitrosourea (MNU)-induced rat mammary adenocarcinoma in vivo

**Figure 2:**
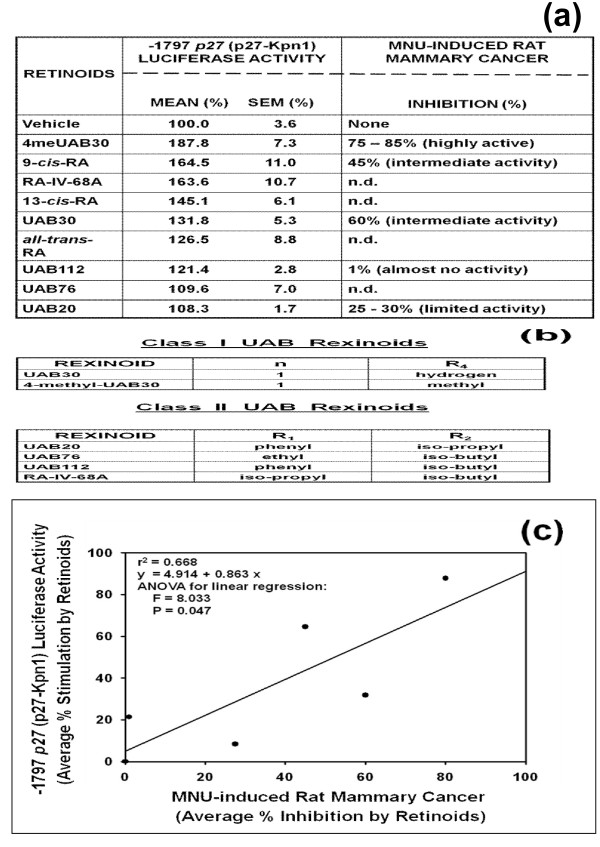
**The degree of up-regulation of p27 expression in human breast cancer cells in vitro linearly correlates with the degree of inhibition of methylnitrosourea (MNU) -induced rat mammary adenocarcinoma in vivo**. (a) Various chemically synthesized retinoic acids were used to investigate whether the degree of up-regulation of -1797 *p27 *gene-luciferase reporter (p27-Kpn I) activity in estrogen receptor (ER)-negative MDA-MB-231 human breast cancer cells *in vitro *correlates with the degree of inhibition of methylnitrourea (MNU) -induced rat mammary adenocarcinoma *in vivo *[33]. The cells were exposed to 1 μM each of the retinoic acids for 24 hours. *In vitro *transfection assays were performed in triplicates and repeated three times. (b) Chemical structure of the retinoic acids used in this experiment [33]. (c) Graphical representation of the results in Figure 2a above.

In the next experiment, we used various chemically synthesized retinoic acids to investigate whether the degree of up-regulation of the -1797 p27-luciferase reporter activity (p27-Kpn I) in human breast cancer cells *in vitro *correlates with the degree of inhibition of methylnitrourea (MNU)-induced rat mammary adenocarcinoma *in vivo*. The results presented in the Figures 2a and 2b indicated that the up-regulation of the *in vitro *p27-luciferase reporter activity by various retinoic acids indeed correlated with the *in vivo *activity of the inhibition of MNU-induced rat mammary cancer by the same retinoic acids [33]. The Figure 2c graphically represents the results in Figure 2a; it shows that the *in vitro *and *in vivo *parameters of the inhibition of breast cancer linearly correlated with each other and the correlation is statistically significant. One note of caution about this linear correlation: if a particular anti-cancer agent (e.g., tamoxifen) needs to be metabolized into an ultimately active anti-cancer agent (e.g., 4-hydroxytamoxifen) *in vivo*, then the *in vitro *and *in vivo *activities of this particular anti-cancer agent (e.g., tamoxifen) do not follow this linear correlation.

### Deletion analysis suggested that 4-hydroxytamoxifen, dexamethasone, all-trans-retinoic acid and 9-cis-retinoic acid activated the proximal 5'-upstream region (-1797) of p27 gene through its 5'-untranslated region (5'-UTR) (-575)

**Figure 3:**
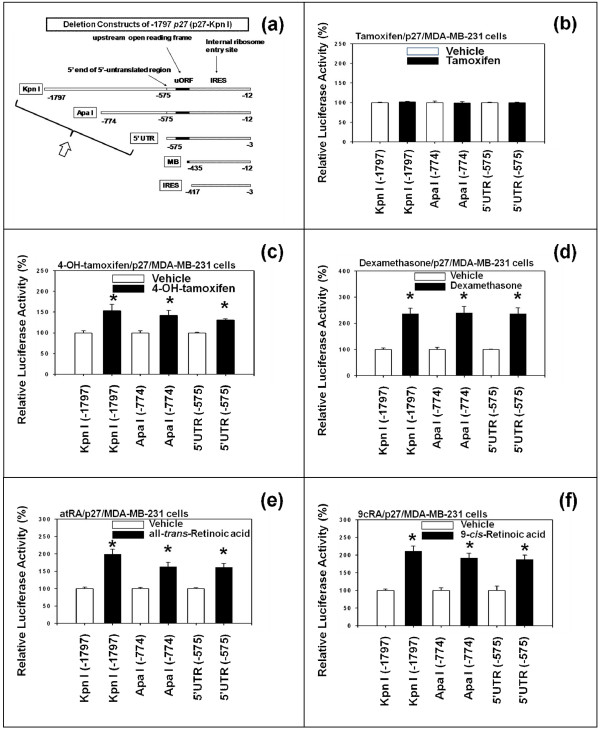
**Deletion analysis indicates that 4-hydroxytamoxifen, dexamethasone, all-trans-retinoic acid (atRA) and 9-cis-retinoic acid (9cRA) activate the proximal 5'-upstream region (-1797) of p27 gene through its 5'-untranslated region (5'-UTR) (-575)**. (a) The 5'-deletion mutants of -1797 *p27 *(p27-Kip I) used in this experiment were -774 *p27 *(p27-Apa I) and -575 *p27 *(p27-5'UTR). Two additional deletion mutants, -435 *p27 *(p27-MB) and -417 *p27 *(p27-IRES), were also used in this experiments, but the data are not shown. The estrogen receptor (ER)-negative MDA-MB-231 human breast cancer cells were transfected with these deletion mutants and then exposed to 1 μM each of (b) tamoxifen, (c) 4-hydroxytamoxifen (4-OH-tamoxifen), (d) dexamethasone, (e) all-*trans*-retinoic acid (atRA) and (f) 9-*cis*-retinoic acid (9cRA) for 24 hours. All assays were performed in triplicates and the transfection experiments were repeated three times.

To determine the core activation elements in the proximal 5'-upstream region (-1797) of *p27 *gene, ER-negative MDA-MB-231 human breast cancer cells were transfected with the following deletion mutants of -1797 *p27 *(Figure 3a): namely -1797 *p27 *(p27-Kpn I) [32], -774 *p27 *(p27-Apa I) [32] and -575 *p27 *(p27-5'-UTR) [11,34]. The transfected cells were then treated with tamoxifen (Figure 3b), 4-hydroxytamoxifen (Figure 3c), dexamethasone (Figure 3d), all-*trans*-retinoic acid (atRA) (Figure 3e) and 9-*cis*-retinoic acid (9cRA) (Figure 3f).

The results suggested that 4-hydroxytamoxifen (but not tamoxifen), dexamethasone, all-*trans*-retinoic acid (atRA), and 9-*cis*-retinoic acid (9cRA) activated proximal 5'-upstream region (-1797) of the *p27 *gene through -575 *p27 *(5'-untranslated region (5'-UTR) of *p27 *gene). When the regions shorter than -575 *p27 *(p27-5'UTR) - namely -435 *p27 *(p27-MB) [32] and -417 *p27 *(p27-IRES) [11,34] - were tested, the activities tended to be either reduced or stay more or less constant (data not shown).

### The -575 p27 (5'-untranslated region (5'-UTR) of p27 gene) was unlikely to contain any cryptic transcription factor binding sites

**Figure 4:**
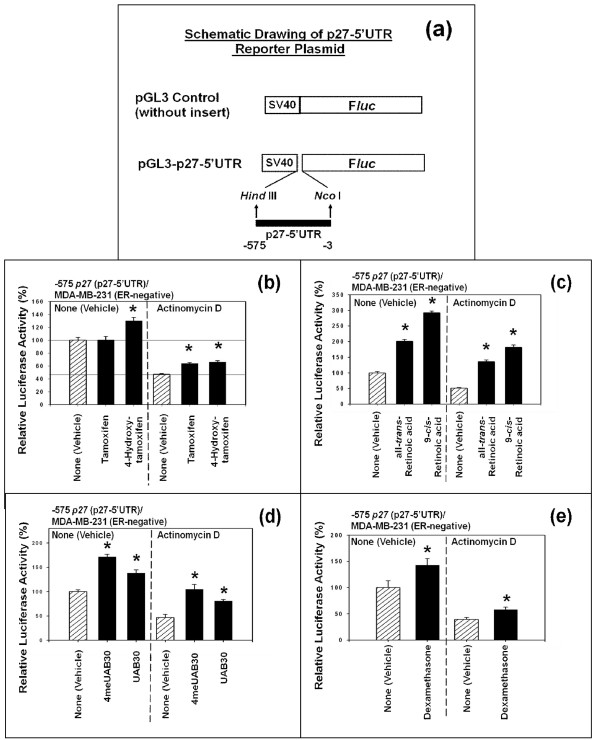
**The 5'-untranslated region (5'-UTR) (-575) of p27 gene is unlikely to contain cryptic transcription factor binding sites**. (a) Schematic drawing (adapted from the references [11,34]) of the pGL3-control-p27-5'-UTR-luciferase reporter plasmid. (b) The ER-negative MDA-MB 231 cells were transfected with -575 *p27 *(p27-5'-UTR) -luciferase reporter plasmid and then treated with either vehicle or actinomycin D(0.5 μg/ml) [55]. One hour after the addition of either vehicle or actinomycin D, the cells were exposed to vehicle, tamoxifen (1 μM) or 4-hydroxytamoxifen (1 μM) for another 24 hours. (c) Same as in Figure 4b above, except that the cells were exposed to vehicle, all-*trans*-retinoic acid (atRA) (1 μM) or 9-cis-retinoic acid (9cRA) (1 μM) for 24 hours. (d) Same as in Figure 4b above, except that the cells were exposed to vehicle, 4-methyl-UAB30 (4meUAB30) (1 μM) or UAB30 (1 μM) for 24 hours. (e) Same as in Figure 4b, except that the cells were exposed to vehicle or dexamethasone (1 μM). All assays were performed in triplicates and the transfection experiments were repeated three times.

To investigate whether -575 *p27 *(p27-5'-UTR) contained any cryptic transcription factor binding sites, the luciferase activity of the 5'-untranslated region (5'-UTR) (-575) of *p27 *gene (p27-5'-UTR) was stimulated by tamoxifen, 4-hydroxytamoxifen, all-*trans*-retinoic acid (atRA), 9-*cis*-retinoic acid (9cRA), UAB30 [33], 4-methyl-UAB30 (4meUAB30) [33], or dexamethasone in the presence and absence of the antibiotic actinomycin D, an inhibitor of transcription. The diagram in Figure 4a shows the schematic drawing of the pGL3-control luciferase reporter plasmid without insert and with p27-5'-UTR insert used for this study. This plasmid - pGL3 control - contained SV40 promoter in its backbone. The preliminary study using pGL3 control without p27-5'-UTR insert had demonstrated that none of the agents or vehicle (DMSO) did not exert any spurious effects on the SV40 promoter when human breast cancer cell lines were used.

The results shown in the left half of the Figure 4b indicated that, in the absence of actinomycin D, only 4-hydroxytamoxifen up-regulated the p27-luciferase activity of -575 *p27 *(p27-5'-UTR) significantly above that of vehicle (DMSO) in MDA-MB-231 cells; as expected, tamoxifen failed to up-regulated it. The results shown in the right half of the Figure 4b indicated that the addition of actinomycin D in the presence of vehicle (DMSO) alone decreased the baseline p27-luciferase activity of -575 *p27 *(p27-5'-UTR) by about 50% compared to the baseline luciferase activity observed in the absence of actinomycin D. Despite this decrease in the baseline p27-luciferase activity in the presence of actinomycin D, 4-hydroxytamoxifen significantly up-regulated the p27-luciferase activity of -575 *p27 *(p27-5'-UTR) above that of the vehicle (DMSO) in the presence of actinomycin D. These results suggested that the transcriptional mechanisms were not involved in a significant manner in the up-regulation of the luciferase activity of -575 *p27 *(p27-5'-UTR) by 4-hydroxytamoxifen, precluding the involvement of any cryptic transcription factor binding sites in this region. What was more surprising was the finding that tamoxifen, which had previously been inactive in the absence of actinomycin D, now significantly up-regulated the p27-luciferase activity of -575 *p27 *(p27-5'-UTR) in the presence of actinomycin D, suggesting that the overall rate of global transcription might somehow exerted effects on the p27-luciferase activity of -575 *p27 *(p27-5'-UTR) in MDA-MB-231 cells.

Similar results were obtained with all-*trans*-retinoic acid (atRA) and 9-*cis*-retinoic acid (9cRA) (Figure 4c), 4-methyl-UAB30 (4meUAB30) [33] and UAB30 [33] (Figure 4d) and dexamethasone (Figure 4e). These results suggested that -575 *p27 *(5'-untranslated region (5'-UTR) of *p27 *gene) was unlikely to have contained any cryptic transcription factor binding sites.

In summary, these results suggested that 4-hydroxytamoxifen, dexamethasone and various retinoic acids up-regulated the expression of p27 by activating translation, rather than transcription, of *p27 *gene via its 5'-untranslated region (5'-UTR) (-575).

### 4-Hydroxytamoxifen and dexamethasone up-regulated the expression of p27 by down-regulating 4E-BP1 phosphorylated at Ser65 and this down-regulation was likely to be mediated by upstream RTKs/Akt/AMPK/mTOR protein kinase signaling pathways. Retinoic acids also up-regulated the expression of p27 but they did so without using any of these pathways

**Figure 5:**
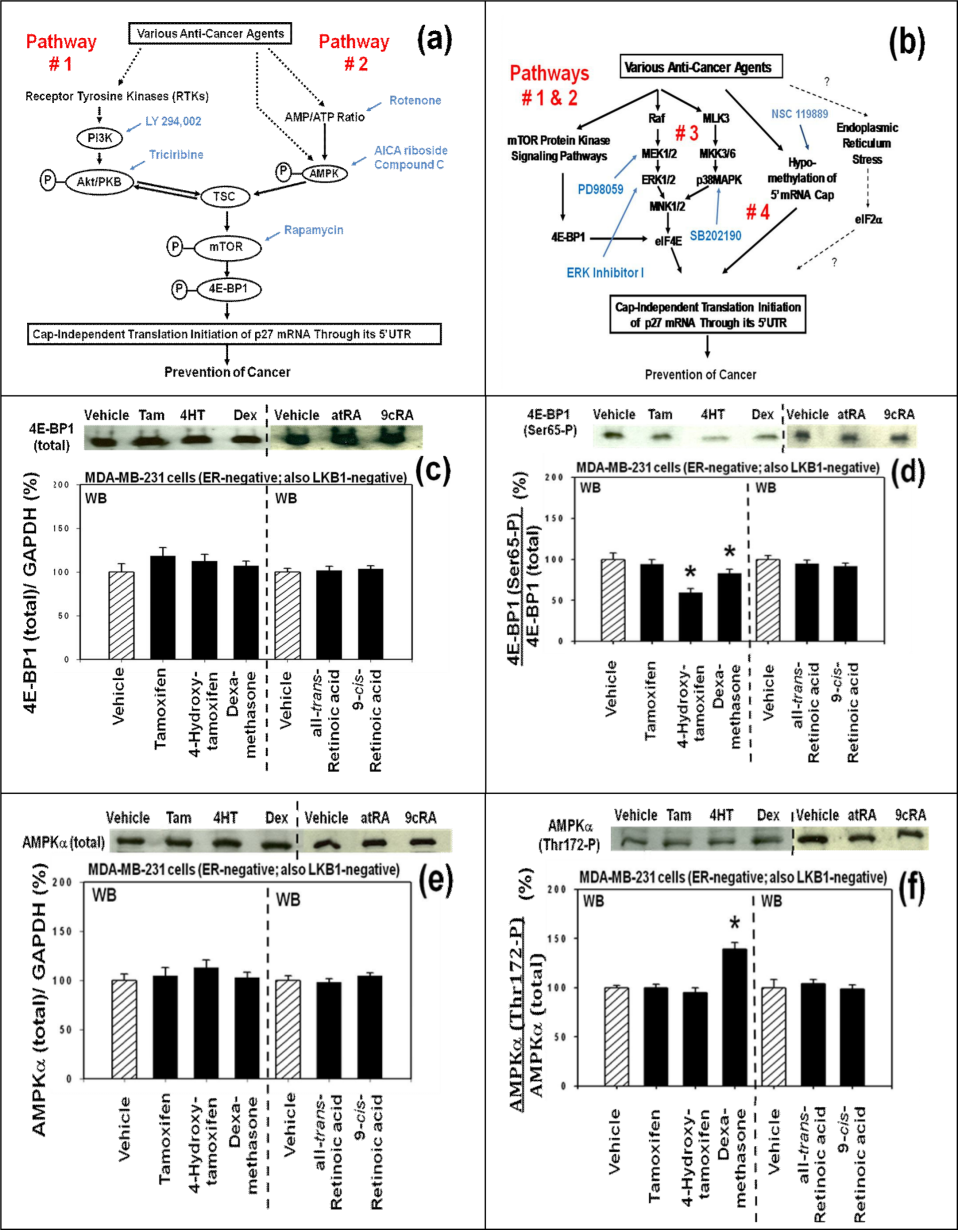
**4-Hydroxytamoxifen and dexamethasone up-regulate the expression of p27 by down-regulating phosphorylation of 4E-BP1 and this down-regulation is likely to be mediated by upstream Akt/AMPK/mTOR protein kinase signaling pathways**. **Retinoic acids also up-regulate the expression of p27 but they do so without using any of these pathways**. (a) and (b): Schematic drawings of the four upstream molecular signaling pathways of p27 expression identified in our previous study [10]. These pathways are: pathway #1 (Figures 5a and 5b), pathway #2 (Figures 5a and 5b), pathway #3 (Figure 5b) and pathway #4 (Figure 5b). The specific inhibitors and activators used previously to identify these four pathways are indicated next to each of the four pathways. From (c) to (f): Estrogen receptor (ER) -negative MDA-MB-231 human breast cancer cells *in vitro *were exposed to vehicle, tamoxifen (1 μM), 4-hydroxytamoxifen (1 μM), dexamethasone (1 μM), all-*trans*-retinoic acid (atRA) (1 μM) or 9-*cis*-retinoic acid (9cRA) (1 μM) for 24 hours. Western immunoblot assays of the cells exposed to these anti-cancer agents were performed using antibodies against (c) total 4E-BP1, (d) 4E-BP1 phosphorylated at Ser65, (e) total AMPK, and (f) AMPK phosphorylated at Thr172. All assays were performed in triplicates and repeated three times. Abbreviations: RTK, receptor tyrosine kinase; PI3K, phosphoinositide 3-kinase; PKB, protein kinase B; AMPK, 5'-AMP-activated protein kinase; TSC, tuberous sclerosis complex; mTOR, mammalian target of rapamycin; eIF4E, eukaryotic translation initiation factor 4E; 4E-BP1, eIF4E-binding protein 1; MAPK, mitogen-activated protein kinase; Raf, MAP kinase kinase kinase; MEK, MAP kinase kinase; MKK, MAP kinase kinase; MNK, MAP kinase-interacting kinase; eIF2α, eukaryotic translation initiation factor 2α.

**Figure 6:**
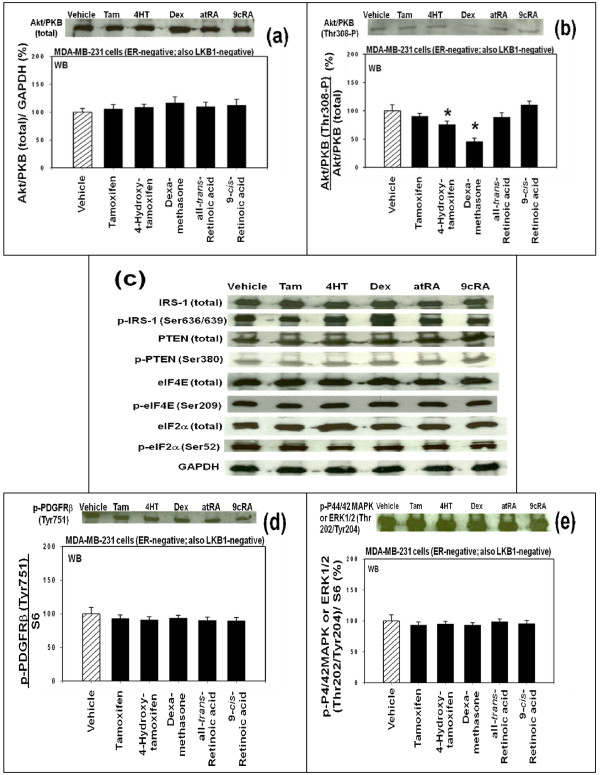
**Further results (continuation of Figure 5) of the Western immunoblot analysis**. Same as in Figure 5, except that Western immunoblot assays of the cells were performed using antibodies against (a) total Akt/PKB, (b) Akt/PKB phosphorylated at Thr308, (c) total IRS-1 (insulin receptor substrate 1), IRS-1 phosphorylated at Ser636/639, total PTEN (phosphatase and tensin homolog), PTEN phosphorylated at Ser380, total eIF4E (eukaryotic translation initiation factor 4E), eIF4E phosphorylated at Ser209, total eIF2α (eukaryotic translation initiation factor 2α), and eIF2α phosphorylated at Ser52, (d) PDGFRβ (platelet-derived growth factor receptor b) phosphorylated at Tyr751, and (e) p44/42 MAPK or ERK1/2 phosphorylated at Thr202Tyr204. All assays were performed in triplicates and repeated three times.

Previous study identified four potential upstream molecular signaling pathways that might be involved in the up-regulation of the expression of p27 by these anti-cancer agents in the ER-negative MDA-MB-231 human breast cancer cells *in vitro *[10]. These four potential upstream molecular signaling pathways of p27 were pathway #1 (Figures 5a and 5b), pathway #2 (Figures 5a and 5b), pathway #3 (Figure 5b) and pathway #4 (Figure 5b). To investigate which one of these upstream molecular signaling pathways was used by 4-hydroxitamoxifen, dexamethasone, all-*trans*-retinoic acid (atRA) and 9-*cis*-retinoic acid (9cRA) to up-regulate the expression of p27, Western immunoblot analysis was performed using the ER-negative MDA-MB-231 human breast cancer cells *in vitro *(Figures 5 and 6). We investigated only the pathways #1, #2 and #3 in this Western immunoblot study; the pathway #4 was not investigated.

Most notable result of this Western immunoblot study was the expression of eukaryotic translation initiation repressor protein 4E-BP1 (eukaryotic translation initiation factor 4E-binding protein 1) phosphorylated at Ser65. As the results in Figure 5c indicate, expression of total 4E-BP1 was neither up nor down-regulated by any of the anti-cancer agents tested (Figure 5c). However, the 4E-BP1 phosphorylated at Ser65 was significantly down-regulated by two of the anti-cancer agents tested, namely 4-hydroxytamoxifen and dexamethasone (Figure 5d). The 4E-BP1 phosphorylated at Ser65 was neither up nor down-regulated by tamoxifen, all-*trans*-retinoic acid (atRA) or 9-*cis*-retinoic acid (9cRA) (Figure 5d). These results suggested that 4-hydroxytamoxifen (but not tamoxifen) and dexamethasone used either the upstream molecular signaling pathway #1 or #2 or both to up-regulate the expression of p27. They also suggested that the two retinoic acids tested did not use pathways#1 and #2 to up-regulate the expression of p27.

The second most notable result of this study was the expression of the following two proteins which were significantly up or down-regulated by one or more of these anti-cancer agents tested: (a) one was AMPKα (5'-AMP-activated protein kinase α) phosphorylated at Thr172 (Figure 5f) and (b) another was Akt/PKB phosphorylated at Thr308 (Figure 6b).

**Figure 7:**
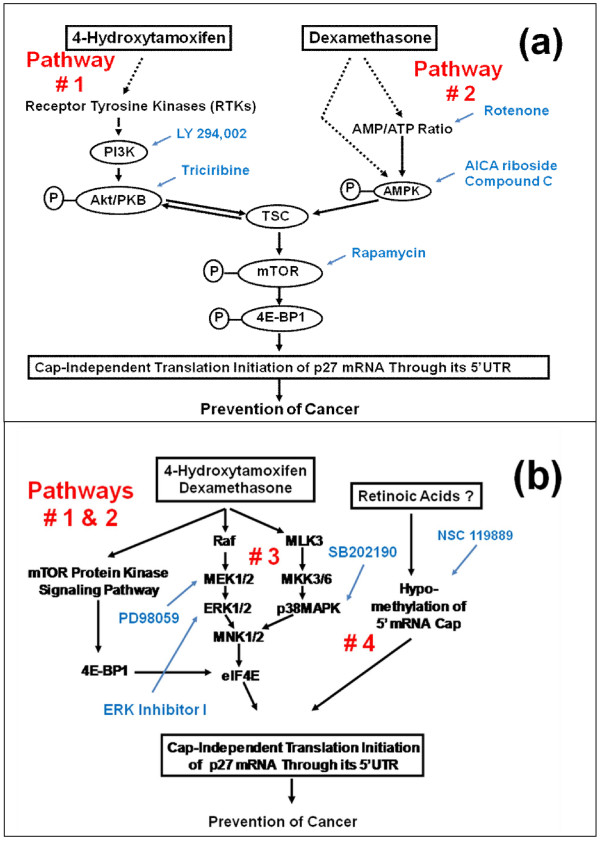
**Schematic drawing of the four upstream molecular signaling pathways of p27 expression that could lead to activation of the unusually long 5'-untranslated region (5'-UTR) (-575) of p27 mRNA by 4-hydroxytamoxifen, dexamethasone and retinoic acids**. (a) The two upstream molecular signaling pathways of p27 shown in Figure 5a are pathways #1 and #2. The pathway #1 consisted of receptor tyrosine kinases/phosphoinositide 3-kinase/Akt/tuberous sclerosis complex/mammalian target of rapamycin/eukaryotic translation initiation factor 4E (eIF4E) -binding protein 1 (RTKs/PI3K/Akt/TSC/mTOR/4E-BP1). The pathway #2 consisted of 5'-AMP-activated protein kinase (metabolic energy sensor or cellular fuel gauge)/tuberous sclerosis complex/mammalian target of rapamycin/eIF4E-binding protein 1 (AMPK/TSC/mTOR/4E-BP1). (b) In addition to these two pathways, two more upstream molecular signaling pathways of p27 expression were previously identified. They were pathways #3 and #4. The pathway #3 consisted of receptor tyrosine kinases/MAPKs/eIF4E (RTKs/MAPKs/eIF4E). The pathway #4 consisted of global hypomethylation of the 5'-7-methylguanosine (m^7^G) cap of mRNAs. The specific inhibitors and activators used previously to identify these four pathways are indicated next to each of the four pathways. The results of this study suggested that 4-hydroxytamoxifen used pathway #1 and dexamethasone primarily used pathway #2 to up-regulate the expression of p27. Dexamethasone could also use a portion of pathway #1 secondarily. We also believe, but could not conclude, that 4-hydroxytamoxifen up-regulated the expression of p27 using MAP kinase pathways (Pathway #3 in Figures 5b and 7b). Retinoic acids up-regulated p27 expression without using pathways #1, #2 and #3. We propose a hypothesis that retinoic acids are likely to have used pathway #4 to up-regulate the expression of p27. Abbreviations: see the legend of Figure 5.

(a) In the case of AMPKα, expression of total AMPKα was neither up nor down-regulated by any of the anti-cancer agents tested (Figure 5e), but the expression of AMPKα phsophorylated at Thr172 was up-regulated by dexamethasone (Figure 5f). Therefore, it is reasonable to assume that dexamethasone up-regulated the expression of p27 by using upstream molecular signaling pathway #2 (Figures 5a, 5b and 7).

(b) In the case of Akt/PKB, expression of total Akt/PKB was neither up nor down-regulated by any of the anti-cancer agents tested (Figure 6a), but the expression of Akt/PKB phosphorylated at Thr308 was down-regulated by 4-hydroxytamoxifen and dexamethasone (Figure 6b). Since 4-hydroxytamoxifen did not up-regulate the expression of AMPKα phosphorylated at Thr172 (Figure 5f), it is likely that 4-hydroxytamoxifen used the upstream molecular signaling pathway #1 exclusively to up-regulate the expression of p27 (Figures 5a, 5b and 7). As for dexamethasone, expression of p27 could have been up-regulated by dexamethasone using either one or both of the following two pathways: namely either (a) dexamethasone used both pathways #1 and #2, or (b) dexamethasone primarily up-regulated AMPKα phosphorylated at Thr172 (pathway #2 in Figures 5a, 5b and 7), the up-regulation of which could have in turn secondarily down-regulated the Akt/PKB phosphorylated at Thr308 (pathway #1 in Figures 5a, 5b and 7).

The third notable result was that all-*trans*-retinoic acid (atRA) and 9-*cis*-retinoic acid (9cRA) neither up nor down-regulated AMPKα phosphorylated at Thr172 (Figure 5f) and also neither up nor down-regulated Akt/PKB phosphorylated at Thr308 (Figure 6b).

Expression of all other proteins examined by Western immuno blot analysis was neither up nor down-regulated by any of the anti-cancer agents tested. The proteins examined in this study included (a) those in the upstream molecular signaling pathway #1 of p27 expression (i.e., total IRS-1 (insulin receptor substrate 1) (Figure 6c), IRS-1 phosphorylated at Ser636/639 (Figure 6c), PDGFRβ (platelet-derived growth factor receptor b) phosphorylated at Tyr751 (Figure 6d), total PTEN (phosphatase and tensin homolog) (Figure 6c), PTEN phosphorylated at Ser380 (Figure 6c)), (b) those in the pathway #3 of p27 expression (p44/42 MAPK or ERK1/2 phosphorylated at Thr202Tyr204 (Figure 6e), total eIF4E (eukaryotic translation initiation factor 4E) (Figure 6c), eIF4E phosphorylated at Ser209 (Figure 6c)) and (c) those involved in the endoplasmic reticulum stress (total eIF2α (eukaryotic translation initiation factor 2α)) (Figure 6c) and eIF2α phosphorylated at Ser52 (Figure 6c)).

## Discussion

The cell cycle repressor protein p27 exhibits a set of unique characteristics that are not seen in other G1-to-S phase cell cycle regulatory proteins including p21 [10]. First, a relatively large number of nutritional and chemopreventive anti-cancer agents specifically up-regulate the expression of p27 without directly affecting expression of other G1-to-S phase cell cycle regulatory proteins. Secondly, the degree of up-regulation of the expression of p27 by these anti-cancer agents in human breast cancer cell lines *in vitro *linearly and positively correlates with the degree of inhibition of methylnitrosourea (MNU)-induced rat mammary adenocarcinoma by the same anti-cancer agents. If a particular anti-cancer agent must be converted to an active metabolite *in vivo *to up-regulate the expression of p27, the degree of up-regulation of p27 *in vitro *and the degree of inhibition of MNU-induced rat mammary adenocarcinoma *in vivo *by the same anti-cancer agent do not follow this linear relationship. An example of such anti-cancer agent is tamoxifen which must be converted to 4-hydroxytamoxifen *in vivo *to up-regulate the expression of p27. Lastly, unlike other G1-to-S phase cell cycle regulatory proteins, expression of p27 is regulated primarily at the level of translation, not at the level of transcription. In the 1980s and 1990s, it was observed that, during the progression of cell cycle, the level of p27 protein expression oscillated cyclically, but the level of p27 mRNA remained constant. This observation led investigators to suggest that, during the cell cycle, expression of p27 is regulated primarily at the level of translation, not at the level of transcription [11,17-19]. The expression of p27 during the cell cycle could also be regulated by various post-translational mechanisms including ubiquitin-proteasome-induced degradation [20-23], complex formation [24], subcellular localization [25-30] and phosphorylation [12,30,31]. Based on the results of our present and previous studies [10], we believe that a relatively large number of nutritional and chemopreventive anti-cancer agents up-regulate the expression of p27primarily by activating the rate of translation.

### 4-Hydroxytamoxifen (but not tamoxifen) up-regulates p27 expression by down-regulating eukaryotic translation initiation repressor protein 4E-BP1 phosphorylated at Ser65 and this down-regulation is likely to be mediated by upstream receptor tyrosine kinases/phosphoinositide-3-kinase/Akt/tuberous sclerosis complex/mammalian target of rapamycin (RTKs/PI3K/Akt/TSC/mTOR) protein kinase signaling pathway (pathway #1)

4-Hydroxytamoxifen (but not tamoxifen) up-regulated expression of p27 in estrogen receptor (ER) -positive as well as negative breast cancer cells *in vitro *[also see the reference [10], suggesting that 4-hydroxytamoxifen up-regulates the expression of p27 regardless of the status of estrogen receptor in the breast cancer cells.

The results also indicated that 4-hydroxytamoxifen (but not tamoxifen) down-regulates eukaryotic translation initiation repressor protein 4E-BP1 phosphorylated at Ser65. It was reported in 2001 that co-expression of the mutant 4E-BP1, which was altered at five different amino acid positions that are normally the targets for phosphorylation, up-regulated the expression of p27 through 5'-untranslated region (5'-UTR) in the proximal upstream region of *p27 *gene in D6P2T Schwannoma cells [35]. Based on this observation and our results taken as a whole, we conclude that down-regulation of 4E-BP1 phosphorylated at Ser65 constitutes an essential component of the upstream molecular signaling pathways of the up-regulation of p27 expression induced by 4-hydroxytamoxifen.

**Figure 8:**
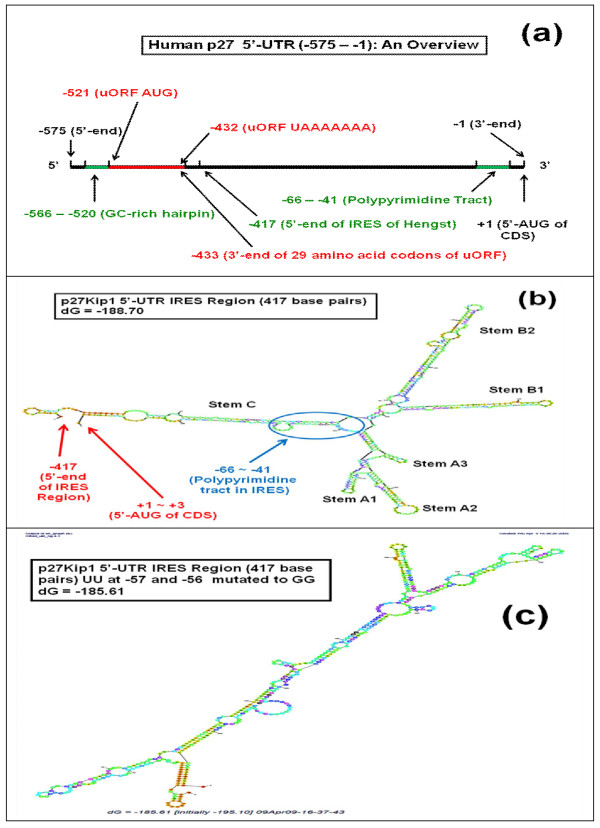
**Diagrams showing the overall primary and secondary structures of the 5'-untranslated region (5'-UTR) (-575) of human p27 mRNA**. (a) Overall primary structure of human 5'-UTR of *p27 *gene showing the location of upstream open reading frame (uORF) (-521 to -425) and the polypyrimidine tract (-66 to -41) in the internal ribosome entry site (IRES) (i.e., IRES region of Hengst: -417 to -1) [11,34]. (b) The most stable secondary stem and loop structure of the wild-type IRES region of Hengst (-417 to -1) [11,34-36] at several lowest Gibbs free energy values. This structure was generated using Zucker's RNA mfold software version 2.3 [56,57]. (c) Same as in Figure 8b, except that UU at -57 and -56 in the polypyrimidine tract (-66 to -41) were mutated to GG. This mutation completely destroyed the overall secondary stem and loop structure of IRES. Various other mutations introduced into the polypyrimidine tract also significantly modified the overall stem and loop structure of IRES.

It is worth noting in this respect that decreased phosphorylation of 4E-BP1 normally leads to decreased translation initiation of mRNAs in general, but for p27 the effect is opposite; it leads, instead, to increased translation initiation of p27 mRNA. This opposite effect of phosphorylated 4E-BP1 on p27 translation initiation is likely to be achieved through its unusually long 5'-untranslated region (5'-UTR) (-575) in the *p27 *gene [11,34-36], which contains two unusual nucleotide motifs, namely uORF (upstream open reading frame) and IRES (internal ribosome entry site) (Figure 8a). Combination of these two elements makes it possible for p27 mRNA to achieve the reverse, cap-independent translation initiation mechanisms as opposed to the normal, cap-dependent translation initiation mechanisms of mRNAs in general.

The critical nucleotide sequence within the IRES motif in the 5'-untranslated region (UTR) of the *p27 *gene resides in the polypyrimidine tract located between -66 and -41 relative to the translation initiation start site (Figures 8a and 8b) [11,34-36]. If this polypyrimidine tract is disrupted by mutations, expression of p27 significantly decreases due to the failure of 40S ribosomal subunit to recognize and bind to the IRES motif (see, for example, Figure 8c). In 2005, an article was published in which the authors induced two mutations in what was called "FOXO response element" located at around -57 relative to the translation initiation start site of *p27 *gene. They observed that these mutations significantly reduced the p27 promoter activity and stated that the disruption of this putative FOXO responsive element decreased the "transactivation of the FOXO response element that was present in the p27 promoter" [37]. Unfortunately, they did not perform gel shift assay to investigate if any transcription factor binds to this element. We believe, along with Koff, Miskimins, Hengst and other investigators [11,34-36], that the transcription of human *p27 *gene starts significantly upstream of -51 - in fact it is -575 in human *p27 *gene - rather than somewhere between -51 and -1 from the translation initiation start site. The so-called FOXO responsive element located at around -51 seems to represent the polypyrimidine tract within the IRES motif which is located between -66 and -41 relative to the translation initiation site.

As for the issue of the upstream molecular signaling pathways of how 4-hydroxytamoxifen up-regulates the expression of p27, 4-hydroxytamoxifen seems to down-regulate phosphorylated 4E-BP1 using upstream receptor tyrosine kinase/phosphoinositide-3-kinase/Akt/tuberous sclerosis complex/mammalian target of rapamycin (RTKs/PI3K/Akt/TSC/mTOR) protein kinase signaling pathway (Pathway #1 in Figure 5a, 5b and 7). This is based on the observation that 4-hydroxytamoxifen down-regulated Akt/PKB phosphorylated at Thr308 without up-regulating AMPKα phosphorylated at Thr172. The results of our previous study also indicated that inhibitors of several receptor tyrosine kinases [see also, for example, reference [38], LY294,002 (inhibitor of PI3K), triciribine (inhibitor of Akt/PKB), and rapamycin (inhibitor of mTOR) up-regulated the p27-luciferase reporter activity in estrogen receptor (ER) -negative MDA-MB-231 human breast cancer cells *in vitro *[10]. We also believe, but could not conclude, that 4-hydroxytamoxifen up-regulated the expression of p27 via MAP kinase pathways (Pathway #3 in Figures 5b and 7b).

### Dexamethasone up-regulates the expression of p27 by down-regulating phosphorylated eukaryotic translation initiation repressor protein 4E-BP1 and this down-regulation is likely to be mediated - primarily - by upstream 5'-AMP-activated protein kinase/tuberous sclerosis complex/mammalian target of rapamycin (AMPK/TSC/mTOR) protein kinase signaling pathway (pathway #2)

Similar to 4-hydroxytamoxifen, dexamethasone also up-regulated the expression of p27 in estrogen receptor (ER) - positive as well as negative breast cancer cells *in vitro *[also see the reference [10]. In addition, dexamethasone down-regulated eukaryotic translation initiation repressor protein 4E-BP1 phosphorylated at Ser65 through 5-untranslated region (5'UTR) (-575) in the proximal upstream region of *p27 *gene.

The effect of dexamethasone on p27 expression appears to be somewhat different from the effect of 4-hydroxytamoxifen in terms of the molecular signaling pathway upstream of 4E-BP1 (Pathways #1 and #2 in Figures 5a, 5b and 7). Unlike 4-hydroxytamoxifen, dexamethasone up-regulated AMPKα phosphorylated at Thr172. The results of previous studies published by other investigators seem to agree with our observation (for example, [39-44]). Since MDA-MB-231 human breast cancer cells are negative not only in estrogen receptor (ER), but also in LKB1 (Drosophila par-4 homologue gene), we believe that dexamethasone up-regulated AMPKα phosphorylated at Thr172 without activating LKB1. The up-regulation of AMPKα phosphorylated at Thr172 by dexamethasone probably led to the up-regulation of p27 expression by way of tuberous sclerosis complex (TSC) proteins, mammalian target of rapamycin (mTOR) and 4E-BP1.

It should be noted that the up-regulation of AMPKα phosphyrylated at Thr172 by dexamethasone could indirectly down-regulate Akt/PKB phosphorylated at Thr308.

In summary, we believe that dexamethasone up-regulated the expression of p27 by down-regulating phosphorylated 4E-BP1 and this down-regulation was mediated primarily by 5'-AMP-activated protein kinase α/tuberous sclerosis complex/mammalian target of rapamycin (AMPKα/TSC/mTOR) protein kinase signaling pathway (Pathway #2 in Figures 5a, 5b and 7). The results of our previous study [10] also indicated that AMPK is involved in both up and down-regulation of p27 expression, namely (a) AICAR (aminoimidazole carboxamide riboside; activator of AMPK), (b) rotenone (inhibitor of Complex I in mitochondrial oxidative phosphorylation), and (c) rapamycin (inhibitor of mTOR) up-regulated the expression of p27. In contrast, Compound C (inhibitor of AMPK) down-regulated the expression of p27 in estrogen receptor (ER) -negative MDA-MB-231 human breast cancer cells *in vitro *[10].

Finally, we do not believe that dexamethasone up-regulated expression of p27 using upstream MAP kinase pathways (Pathway #3 in Figures 5b and 7b).

### Retinoic acids also up-regulate the expression of p27 but they do so without using any of the pathways described above for 4-hydroxytamoxifen and dexamethasone

Retinoic acids up-regulated the expression of p27 in human breast cancer cells *in vitro *without down-regulating 4E-BP1 phosphorylated at Ser65. Retinoic acids also did not use upstream molecular signaling pathway #1 (RTKs/PI3K/Akt/TSC/mTOR) (Figures 5a, 5b and 7), #2 (AMPK/TSC/mTOR) (Figures 5a, 5b and 7), or - probably - #3 (MAP kinases) (Figures 5b and 7).

Of the four upstream molecular signaling pathways of p27 expression identified previously, we investigated only three pathways (#1, #2 and #3) in the present study. The pathway #4 was not investigated. Potential involvement of the pathway #4 in the expression of p27 by retinoic acids was suggested by the results of our previous study where NSC 119889, an inhibitor of the global methylation of 5'-m^7^G-cap of mRNAs, up-regulated the p27-luciferase reporter activity of the 5'-untranslated region (5'UTR) (-575) within the proximal upstream region of the *p27 *gene (Figures 5b and 7b) [10].

It is known that nearly all mRNAs are post-transcriptionally modified at their 5' and 3' ends by capping and polyadenylation, respectively [45-47]. The m^7^G-capping at the 5' end protects the nascent pre-mRNAs against degradation. Therefore, failure to cap or loss of cap leads to rapid breakdown of mRNAs. The enzyme 5'-mRNA cap (guanine-N^7^) methyltransferase catalyzes transfer of methyl group from S-adenosylmethionine (AdoMet or SAM) to GpppRNA to form m^7^GpppRNA. We observed in our previous study [10] that NSC 119889, a cell-permeable, competitive inhibitor of AdoMet (SAM), inhibited global cap-dependent translation initiation of 5'-m^7^G-capped mRNAs in general, but it increased cap-independent translation initiation of p27 mRNA through its 5'-UTR in estrogen receptor (ER) -negative MDA-MB-231 human breast cancer cells *in vitro*.

Schalinske and other investigators have been reporting for almost two decades that retinoic acids decrease the ratio of S-adenosylmethionine (AdoMet or SAM) to S-adenosylhomocysteine (AdoHcy or SAH) presumably by inducing glycine N-methyl transferase [48-53]. This observation suggests that retinoic acids decrease the ratio of SAM/SAH thereby inducing global hypomethylation of 5'-m^7^G-cap of mRNAs, which in turn up-regulates the expression of p27 by increasing reverse, cap-independent translation initiation of p27 mRNA through its 5'-UTR.

Based on these considerations, we propose that retinoic acids up-regulate the expression of p27 by reducing the methylation of the 5'-m^7^G -cap of mRNAs in general, and at the same time, increasing the reverse, cap-independent translation initiation of p27 mRNA through its 5'-UTR (pathway #4 in Figures 5b and 7b).

## Conclusions

Based on the results presented above, we conclude that:

(a) 4-Hydroxytamoxifen (but not tamoxifen) up-regulates the expression of p27 in both estrogen receptor-positive and negative human breast cancer cells *in vitro *by down-regulating phosphorylation of 4E-BP1 and this down-regulation is mediated by upstream receptor tyrosine kinases/phosphoinositide 3-kinase/Akt/tuberous sclerosis complex proteins/mammalian target of rapamycin (RTKs/PI3K/Akt/TSC/mTOR) protein kinase signaling pathway (pathway #1 in Figures 5a, 5b and 7). We also believe, but could not conclude, that 4-hydroxytamoxifen up-regulates the expression of p27 using MAP kinase pathways (Pathway #3 in Figures 5b and 7b).

(b) Dexamethasone up-regulates the expression of p27 in both estrogen receptor-positive and negative human breast cancer cells *in vitro *by down-regulating phosphorylation of 4E-BP1 and this down-regulation is mediated primarily by upstream 5'-AMP-activated kinase/tuberous sclerosis complex proteins/mammalian target of rapamycin (AMPK/TSC/mTOR) protein kinase signaling pathway (pathway #2 in Figures 5a, 5b and 7). We do not believe that dexamethasone up-regulated expression of p27 using MAP kinase pathways (Pathway #3 in Figures 5b and 7b)

(c) Retinoic acids also up-regulate the expression of p27 in both estrogen receptor-positive and negative human breast cancer cells *in vitro*, but they do so without using any of the pathways described above for 4-hydroxytamoxifen and dexamethasone. We propose that retinoic acids up-regulate the expression of p27 by decreasing the ratio of SAM/SAH thereby inducing hypomethylation of the 5'-m^7^G-cap of p27 mRNA. Hypomethylation of the 5'-cap of p27 mRNA in turn activates the reverse, cap-independent translation initiation of p27 mRNA through its 5'-untranslated region (5'-UTR), which contains upstream open reading frame (uORF) and internal ribosome entry site (IRES) (pathway #4 in Figures 5b and 7b).

## Methods

### Reagents

Tamoxifen, 4-hydroxytamoxifen, dexamethasone, all-*trans*-retinoic acid (atRA), 9-*cis*-retinoic acid (9cRA), 13-*cis*-retinoic acid (13cRA), and actinomycin D, were purchased from Sigma-Aldrich (St. Louis, MO, USA). The following retinoic acids were generously provided by Dr. Muccio [30] at the University of Alabama at Birmingham (Birmingham, AL, USA.): namely 4-methyl-UAB30 (4meUAB30), RA-IV-68A, UAB30, UAB112, UAB76 and UAB20. The chemical structure and cancer chemopreventive activity of atRA, 9cRA, 13cRA, and other retinoic acids in the MNU-induced rat mammary adenocarcinoma in vivo were described in the reference #33.

The following antibodies were purchased from Cell Signaling Technology, Inc. (Danvers, MA, USA): namely (a) total 4E-BP1 and phospho-4E-BP1 (Ser65); (b) total AMPKα and phospho-AMPKα (Thr172); (c) total Akt and phospho-Akt (Thr308); (d) total IRS-1 and phospho-IRS-1 (Ser636/639), (e) phospho-PDGFRβ (Tyr751), (f) total PTEN and phospho-PTEN (Ser380), (g) phospho-p44/42MAPK or ERK1/2 (Thr202/Tyr204), (h) total eIF4E and phospho-eIF4E (Ser209); (i) total eIF2α and phospho-eIF2α (Ser51) and (j) S6. Additionally, the following two antibodies were purchased from Santa Cruz Biotechnology, Inc. (Santa Cruz, CA, USA): namely (a) p27(F-8) and (b) GAPDH.

### Cell Cultures

Human MCF7 (estrogen receptor-positive and LKB1-positive) and MDA-MB-231 (estrogen receptor-negative and LKB1-negative) breast cancer cell lines were purchased from the American Type Culture Collection (Rockville, MD, USA). MCF7 cells were grown in Dulbecco's Modified Eagle's Medium (DMEM) containing 4.5 g/L of D-(+)-glucose, supplemented with 10% heat-inactivated fetal bovine serum (FBS), 100 mg/L recombinant human insulin, 2% L-glutamine, and antibiotic/antimycotic solution. MDA-MB-231 cells were grown in the same culture medium without insulin. The incubation was carried out at 37°C in a 5% CO_2 _humidified chamber. All cells were subcultured after trypsinization with 0.05% trypsin-0.02% EDTA solution. The cell cultures were always maintained below confluency. The cells were checked periodically for mycoplasmal infection by DNA fluorochrome staining.

### Plasmids

Luciferase reporter plasmids containing one of the following proximal 5'-upstream region of the *p27 *gene were used to transfect the human breast cancer cells: -1797 *p27 *(p27-Kpn I) [32], -774 *p27 *(p27-Apa I) [32], -575 *p27 *(p27-5'-UTR) [11,34], -435 *p27 *(p27-MB) [32], and -417 *p27 *(p27-IRES) [11,34] (see Figures 1b and 3a). The control luciferase reporter plasmids that did not contain these inserts were also prepared to test if nutritional and chemopreventive anti-cancer agents were exerting any spurious effects on the backbone rather than the insert of the luciferase reporter plasmids. All of the nutritional and chemopreventive anti-cancer agents tested did not exert spurious effects on the backbone of the luciferase reporter plasmid in the human breast cancer cells *in vitro*.

### Transfection and Luciferase Assay

Transfections were performed according to the published protocol [54] using FuGENE 6 purchased from Roche Applied Science (Indianapolis, IN, USA). Briefly, 24 hours before the transfection of luciferase-reporter plasmid, cells were seeded into a 60-mm tissue culture dish containing 3 mL of Dulbecco's Modified Eagle Medium (DMEM) supplemented with 10% heatinactivated fetal bovine serum (FBS), 2% L-glutamine, and antibiotic/antimycotic solution at a density of 1.5 × 10^5 ^cells/dish and incubated at 37°C in a 5% CO_2 _humidified chamber. Transfection was carried out with 1 μg of luciferase reporter plasmid and 0.2 μg of pSV-β-galactosidase internal control plasmid (Promega, Madison, WI, USA) mixed with 3 μL of FuGENE 6 solution in 3 mL of FBS-free DMEM supplemented with only 2% L-glutamine. A minimum of 5-hour incubation at 37°C was needed for transient transfection, followed by 18-hour incubation in DMEM with 10% FBS for recovery. The transfected cells were then partially synchronized in DMEM with 0.2% FBS for 24 hours. The resulting cells were then treated with various anti-cancer agents in the same culture medium as described in the figure legends. After 24 hours, the treated cells were collected and lysed using Reporter Lysis Buffer (Promega, Madison, WI). The resulting cell lysates were assayed for luciferase activity using Luciferase Assay Kit (Promega, Madison, WI, USA) and TD-20/20 Luminometer (Turner Designs, Sunnyvale, CA, USA). β-Galactosidase activity was measured using chlorophenol red-β-D-galactopyranoside (CPRG) (Sigma-Aldrich, St. Louis, MO, USA) as substrate.

Each luciferase activity driven by a specific proximal 5'-upstream region of the *p27 *gene was normalized to β-galactosidase activity, a control for transfection efficiency. Since certain nutritional and chemopreventive anti-cancer agents could sometimes stimulate the normalized luciferase activity of empty luciferase reporter that do not contain any insert of the proximal 5'-upstream region of the *p27 *gene, a special formula [9,10] was used in these exceptional cases to correct for this false increase in the relative luciferase activity. With human breast cancer cell lines used in this study, we have not encountered any such exceptional cases.

### Western Immunoblot Analysis

Western immunoblot analysis of the upstream molecular signaling pathways of p27 expression was performed using estrogen receptor (ER) -negative MDA-MB-231 human breast cancer cells *in vitro*. The analysis was performed without either transfecting the cells with proximal 5'-upstream region of *p27 *gene-luciferase reporter plasmid or adding growth factors to stimulate the proliferation of the cells.

The cells were first seeded at a density of 5.5 × 10^6 ^cells/dish into a 100-mm tissue culture dish containing 10 mL of DMEM supplemented with 10% heat-inactivated fetal bovine serum (FBS), 2% L-glutamine, and antibiotic/antimycotic solution and incubated at 37°C in a 5% CO_2 _humidified chamber for 24 hours. After 24 hours, the cells were partially synchronized for another 24 hours in DMEM containing 0.2% FBS. Then, the cells in the 0.2% FBS-DMEM culture medium were treated with vehicle (DMSO), tamoxifen, 4-hydroxytamoxifen, dexamethasone, all-*trans*-retinoic acid (atRA), or 9-*cis*-retinoic acid (9cRA) for another 24 hours. After 24 hours, the cells were washed twice with cold 1× PBS and scraped in 1× RIPA Lysis Buffer (Santa Cruz Biotechnology, Santa Cruz, CA, USA) containing phenylmethylsulphonyl fluoride (PMSF), protease inhibitor cocktail and sodium orthovanadate, and supplemented with 50 mM NaF. The cells were then sonicated and the supernatant was collected by centrifugation and stored at -80°C.

The supernatants (50 μg protein/lane) were applied to the SDS-PAGE and, after fractionation, proteins were transferred to nitrocellulose membrane, which was then blocked and incubated in a solution containing first primary antibody. After shaking overnight at 4°C, the target proteins bound to the first primary antibody were further treated with alkaline phosphatase (AP)-conjugated secondary anti-immunoglobulin antibody and detected by chemiluminescence using TROPIX Western-Star Kit (Applied Biosystems, Foster City, CA, U.S.A.). After exposure to X-ray film, the blots were stripped using Western Re-Probe solution (G-Biosciences, St. Louis, MO, U.S.A.), checked for removal of the chemiluminescence and then re-probed with second primary antibody.

Densitometric measurement of the intensity of the bands on the X-ray film was performed using UN-SCAN-IT Gel & Graph Digitizing Software Version 6.1 (Silk Scientific Corporation, Orem, UT, U.S.A.). Background corrections were done by four corner interpolation and optical density calculations were performed using linear standard reflective scan method.

### Statistical Analysis

All of the significant P values were between 0.01 and 0.05. So, the results with P values less than 0.05 are simply indicated as asterisk on top of the vertical bars. The statistical significance information for the regression analysis, however, was provided in more detail in the panel Figure 2c.

## List of abbreviations used

### Nonstandard abbreviations

p27: p27Kip1; p21: p21Cip1/Waf1; AMPK: 5'-AMP-activated protein kinase; TSC: tuberous sclerosis complex; mTOR: mammalian target of rapamycin; RTK: receptor tyrosine kinase; PTEN: phosphatase and tensin homolog; PI3K: phosphoinositide 3-kinase; PKB: protein kinase B; MAPK: mitogen-activated protein kinase; MEK: mitogen-activated protein (MAP) kinase kinase; ERK: ERK MAP kinase; MNK: MAP kinase interacting kinase; m^7^G: 7-methylguanosine; CDK: cyclin-dependent kinase; CDI: cyclin-dependent kinase inhibitor; MNU: *N*-methyl-*N*-nitrosourea; atRA: all-*trans*-retinoic acid; 9cRA: 9-*cis*-retinoic acid; 13cRA: 13-*cis*-retinoic acid; 4meUAB30: 4-methylUAB30; ER: estrogen-receptor; 5'-UTR: 5'-untranslated region; IRES: internal ribosome entry site; DMSO: dimethyl sulfoxide; pGL3: pGL3 luciferase reporter vector; SV40: simian virus 40; EGFR: epidermal growth factor receptor; PDGFR: platelet-derived growth factor receptor; IR: insulin receptor; IGR-1R: type 1 insulin-like growth factor receptor; AdoMet or SAM: S-(5'-adenosyl)-L-methionine; AdoHcy or SAH: S-(5'-adenosyl)-L-homocysteine; eIF2α: eukaryotic translation initiation factor 2α; 4E-BP1: eukaryotic translation initiation factor 4E binding protein 1; S6K: p70 S6 kinase; AICAR: 5-amino-4-imidazolecarboxamide aminoimidazole carboxamide ribonucleotide; Glc: D-(+)-glucose; Ser: L-serine; Thr: L-threonine; Met: L-methionine; Cys: L-cysteine; Leu: L-leucine; Tyr: L-tyrosine; eIF4E: eukaryotic translation initiation factor 4E; uORF: 5'-upstream open reading frame; FBS: fetal bovine serum; DMEM: Dulbecco's modified Eagle's medium; EDTA: ethylenediaminetetraacetic acid; CPRG: chlorophenol red-β-D-galactopyranoside; βGal: β-galactosidase; Luc: firefly luciferase; GAPDH: glyceraldehydes phosphate dehydrogenase.

## Competing interests

The author declares that he has no competing interests.
